# Interdisciplinary collaboration in action: tracking the signal, tracing the noise

**DOI:** 10.1057/palcomms.2015.19

**Published:** 2015-07-21

**Authors:** Felicity Callard, Des Fitzgerald, Angela Woods

**Affiliations:** 1 The Hub at Wellcome Collection, Wellcome Collection, London, UK; 2 Department of Geography, Durham University, Durham, UK; 3 School of Medicine, Pharmacy & Health, Durham University, Durham, UK; 4 Centre for Medical Humanities, Durham University, Durham, UK

## Abstract

Interdisciplinarity is often framed as an unquestioned good within and beyond the academy, one to be encouraged by funders and research institutions alike. And yet there is little research on how interdisciplinary projects actually work—and do not work—in practice, particularly within and across the social sciences and humanities. This article centres on “Hubbub”, the first interdisciplinary 2-year research residency of The Hub at Wellcome Collection, which is investigating rest and its opposites in neuroscience, mental health, the arts and the everyday. The article describes how Hubbub is tracing, capturing and reflecting on practices of interdisciplinarity across its large, dispersed team of collaborators, who work across the social sciences, humanities, arts, mind and brain sciences, and public engagement. We first describe the distinctiveness of Hubbub (a project designed for a particular space, and one in which the arts are not positioned as simply illustrating or disseminating the research of the scientists), and then outline three techniques Hubbub has developed to map interdisciplinary collaboration in the making: (1) ethnographic analysis; (2) “In the Diary Room”, an aesthetics of collaboration designed to harness and capture affective dynamics within a large, complex project; and (3) the Hubbub Collaboration Questionnaire, which yields quantitative and qualitative data, as well as a social network analysis of collaborators. We conclude by considering some themes that other inter-disciplinary projects might draw on for their own logics of tracking and tracing. This article forms part of an ongoing thematic collection dedicated to interdisciplinary research.

## Introduction

Over the last two decades, the hortatory power of interdisciplinarity within and beyond the academy has grown substantially. This is particularly the case across the humanities and social sciences, where, in terms of overt rhetoric, interdisciplinarity had not, until recently, been considered an epistemological norm, as it had in many of the sciences (for example, Lauterbur, 2004).^1^ In the face of this growth, there has not yet been any significant emergence of research *on practices* of interdisciplinarity within the social sciences and humanities. Despite some exceptions (for example, Quan-Haase *et al.*, 2015), we remain largely ignorant, then, of how the heterogeneous subjects, objects and methodologies of interdisciplinarity are created, shaped and transformed in and by their day-to-day lives within distinctive collaborative ecologies.

This article describes a preliminary foray into this arena. It is co-authored by three researchers from “Hubbub”, the first research residency of The Hub at Wellcome Collection (Wellcome Collection, n.d.), a major new location for interdisciplinary research. Hubbub is a large interdisciplinary project, investigating rest and its opposites (tumult, noise, work, exertion) in neuroscience, mental health, the arts and the everyday (Hubbub, 2015); it comprises a team of approximately 50 people from the humanities, social sciences, mind and brain sciences, arts, media and public engagement; the project is based in The Hub at Wellcome Collection from October 2014 till July 2016. Our own disciplinary formations as co-authors have come via geography (Callard), sociology (Fitzgerald), and cultural studies (Woods); across those domains, we are united by our shared interest in conceptualizing and theorizing interdisciplinarity, and by a long history of engaging in interdisciplinary projects (for example, Callard and Kerbel, 2002; Callard and Fitzgerald, forthcoming; Fitzgerald, 2012; Bernini and Woods, 2014; Fitzgerald *et al.*, 2014; Woods *et al.*, 2014; Fernyhough *et al.*, 2015; Fitzgerald and Callard, 2015).

The article has two aims. First, we describe Hubbub and its theatre of operations in an architecturally designed space: The Hub at Wellcome Collection. We itemize some of Hubbub’s distinctive epistemological and ontological starting points, as well as some of the challenges and opportunities in relation to interdisciplinarity afforded both by its structure and by the space in which it is situated. Second, we outline three techniques that we have developed to track, encourage and reflect on interdisciplinarity as practised by Hubbub collaborators within and beyond The Hub. This article comprises one of a number of publications from Hubbub that tracks interdisciplinarity as an object of conceptual and empirical investigation, as a method of working, and as a phenomenon subject to historical and geographical variation. In this early contribution (data will be presented in future publications), we focus on the techniques we have chosen—and which we are currently implementing, as Hubbub moves from set-up to research phase—to map interdisciplinarity. We clarify why we have chosen these particular techniques to understand the textures, affordances and constraints of interdisciplinary working within Hubbub, which we hope will have implications for interdisciplinary working practices elsewhere.

## Hubbub and The Hub

**Figure 1:**
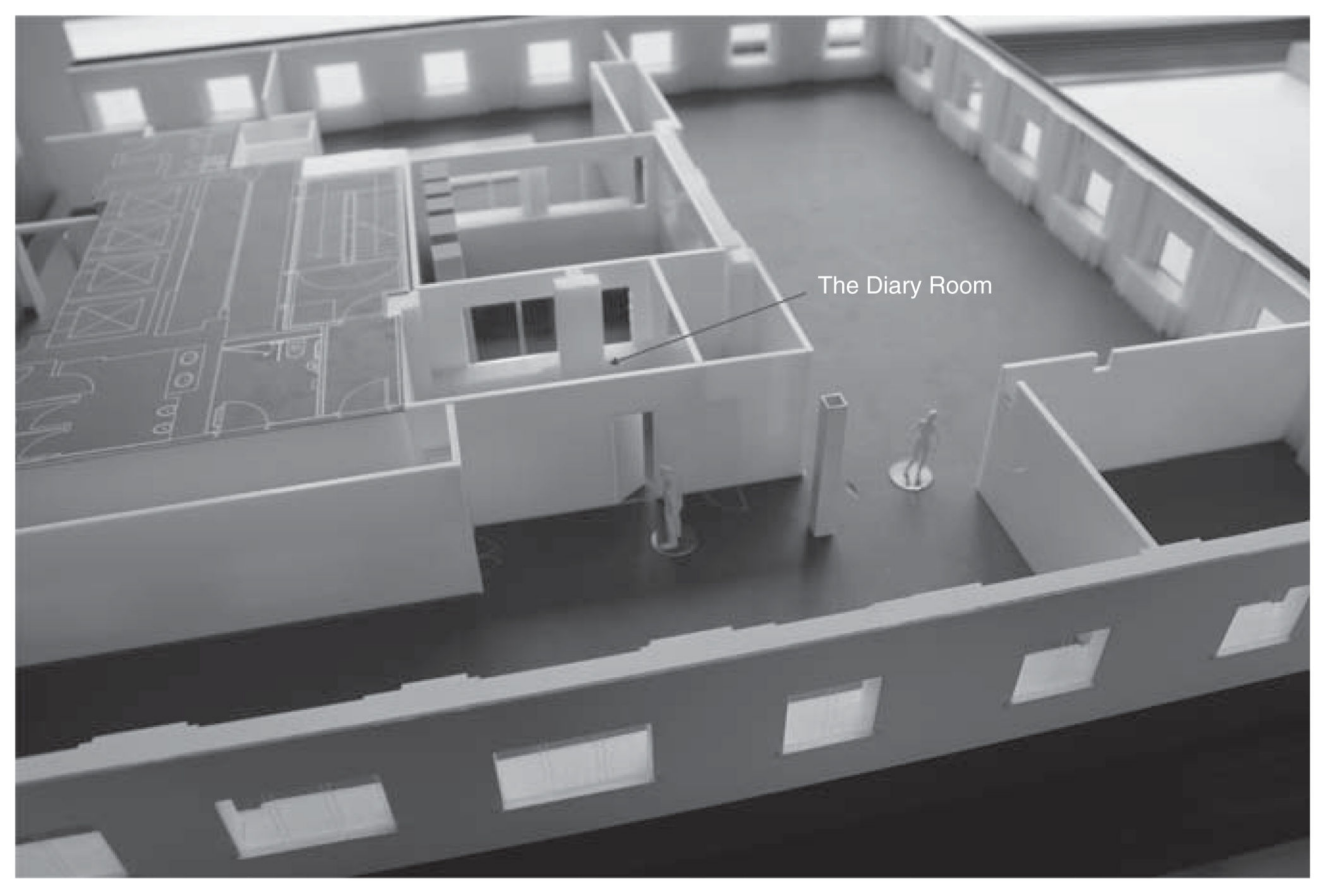
The Hub at Wellcome Collection. The Diary Room (marked) is a private room set off from the open-plan, two-winged space. Architectural model by Wilkinson Eyre Architects; photograph by James Wilkes.

Hubbub emerged in response to a new funding call by The Wellcome Trust for 2-year interdisciplinary residencies. At the centre of the call was The Hub itself, a distinctive physical space (see Fig. 1) and set of resources that have been developed within Wellcome Collection on Euston Road, in the heart of London. Wellcome Collection opened to the public in 2007 to explore interdisciplinary connections between science, art and everyday life through exhibitions, gallery spaces and various kinds of public engagement. The Hub was constructed as part of Wellcome Collection’s major redevelopment (started in 2013, completed in 2015) to create new spaces, a more immersive experience for visitors and more opportunities to experiment (whether via a new studio for young people, via a public reading room, which crosses from the Wellcome Library to the exhibition spaces, or via The Hub). When the redevelopment of Wellcome Collection was announced, The Wellcome Trust described The Hub as “a new space for interdisciplinary research”, which will “catalyse research and public engagement collaborations between the brightest minds across specialisms” (Wellcome Trust, 2012). As both a funding award and a space, The Hub aims to act as “a pioneering location for creative work that explores what happens when medicine and health intersect with the arts, humanities and social sciences” (Wellcome Trust, n.d.).

Hubbub is led by Durham University, with support from the Neuroanatomy and Connectivity Research Group of the Max Planck Institute for Human Cognitive and Brain Sciences; it was selected, through a lengthy competitive process, from 55 applications to take up the first residency of The Hub. Hubbub is led by a Director and Principal Investigator (Callard, a social scientist), with a “Core Group” comprising the Director and four Associate Directors/Co-Investigators (Charles Fernyhough, a psychologist and writer; Claudia Hammond, a writer and BBC broadcaster; Daniel Margulies, a cognitive neuroscientist with a background in the humanities; and James Wilkes, a poet and humanities researcher). Beyond the Core Group, Hubbub is made up of a dense network of over 40 collaborators (who include psychologists, a medieval and a modern historian, a composer, poets and sound-based writers and performers, a curator, cognitive neuroscientists, mental health clinicians and public mental health experts); the majority of collaborators are either freelance or based at institutions across the United Kingdom, Germany and the United States; they pass in and out of the Hub at different frequencies, and for differing lengths of visits/residencies. Hubbub is also supported by a Project Coordinator, while the dedicated Wellcome Hub Partnership Manager liaises between the residency and the Trust.

Hubbub researchers are investigating rest and its opposites at different scales (including those of the brain, mind, body and city), using forms of creative experimentation. Research in progress includes: (1) development of an innovative survey (the “Rest Test”) to track people’s practices of rest within the United Kingdom and beyond (this will be launched through BBC Radio 4); (2) development of an interdisciplinary neuroscience/social science book series (Neuroscience Intersections) to launch in the autumn of 2015; (3) the development of self-tracking tools that capture physical movement, geographical location, as well as phenomenological assessments of activity, rest and the environment as participants move around the city (Berson, forthcoming); (4) collaborative sound works performed across London; (5) new poetry, performance and musical compositions that interrogate noise, sound and silence; (6) scientometric analyses of the sub-fields of “resting state” cognitive neuroscience; (7) the development of interdisciplinary methods to investigate inner experience (for example, Kühn *et al.*, 2014); and (8) conceptual, creative and activist engagements with how rest is being denied to unemployed people who are subject to “workfare” practices (Friedli and Stearn, 2015).

Projects such as these have been taking form through smaller and larger cross-disciplinary groups (some involving 2 people, some 15). Frequently, the Hub is used as the site for intensive working groups and/or “summits” that allow people to work through methodological, conceptual and empirical difficulties together; at other times, the Hub is filled with people quietly writing, thinking and experimenting alone. Hubbub’s work regularly moves outside the Hub both for research and for public engagement events. One collaborator is developing new participatory models through which to capture noise experienced by people living under the Heathrow flight path; other collaborators are taking scientific and artistic installations investigating rest to summer festivals. The project deliberately side-steps the traditional boundaries of professional, scientific and artistic practice, to develop shared methods, sources, data and modes of working.

The fact that Hubbub is physically located *within* its funder has influenced the kinds of interdisciplinary practices that it has developed and that are possible, as well as how it has tried to torque various methods for eliciting and capturing interdisciplinarity. The Wellcome Trust, for its part, has described The Hub as a “grand experiment”, and hopes that there will be many points of intersection and collaboration between researchers undertaking Hub residencies, Trust staff and Wellcome Collection resources (including the Wellcome Library, Archives and Reading Room). It has championed a wide-ranging and experimental approach to interdisciplinarity, one that we see as extending well beyond the integrationist logic that characterizes many such endeavours (for an analysis of this logic, see Fitzgerald and Callard, forthcoming).

In Hubbub, we have deliberately worked to harness, and push on, this approach. For example, most “interdisciplinary” projects that bring the arts and humanities into contact with the sciences tend to stage the arts as in some way “exemplifying” the science or acting as the key mediator or outreach agent for that science (for example, Drumm *et al.*, 2015). Hubbub, by contrast, insists that the arts engage in creative experiments that are just as methodologically and conceptually rigorous, just as generative of knowledge, as those conducted by disciplines not commonly prefaced with the adjective “creative”. It is, furthermore, uncommon for interdisciplinary projects to contain such a concentration of researchers who are themselves preoccupied by the conceptual shape and practical operations of interdisciplinarity (we include, beyond the authors of this article, for example, co-investigators Charles Fernyhough (Fernyhough *et al.*, 2015), Daniel Margulies (co-founder of the Neuro Bureau, n.d.) and James Wilkes (Wilkes and Scott, forthcoming)). Hubbub is thus animated by a series of principles and preoccupations that we take to be central to any serious assessment of interdisciplinarity in practice. Three that we believe to be particularly useful to discuss, in light of the techniques we elaborate on in the latter half of this article, comprise:

- *Spaces of interdisciplinarity*: There is a growing acknowledgement of how central the designing and curating of particular kinds of socio-spatial interaction can be to the practice of interdisciplinarity (Dzeng, 2013). The research literature on this topic is, though, small—and what does exist tends to analyse conceptually how different articulations of space map onto, and are enfolded within, different conceptualizations of interdisciplinarity (for example, Walls, 2011; Fitzgerald and Callard, 2015). The Hub space, by contrast, builds geographical and epistemological dislocation into its basic fabric. A totally open space arcs into corners and edges for shared conceptual or studio practice, while no topological distinctions are granted to different ways of doing intellectual work: soft furnishings intermingle with hard desks; tables can be shared or not; a hammock for lounging in has been installed by Hubbub at one end of the room, even as an all-seeing, all-recording specially built “scriptorium” marks the other.
- *Experimentation*: Hubbub is committed to the idea that practices and histories of experimentation are central to all the domains of expertise that comprise the project. The archives of experimentation within poetry, literature, performance—as well as within the disciplines of the humanities—are as rich and resonant for understanding and working with experimentation as those from the history of science and medicine. Hubbub, therefore, establishes its “artists” on the same field as its “scientists”, even as it has no illusion about discrepancies in institutional power. As we have documented elsewhere, our epistemological and ontological starting points attempt to place aesthetic practices on the same plane as scientific ones, without gainsaying the complex inequities in power and epistemological authority (Callard, 2014; Wilkes, 2014). The point is that Hubbub’s logic of experimentation is very deliberately trying to break the conventionalized gestures of (for example) “SciArt” and affiliate practices—modes that tend to assign distinctive empirical and interpretive roles to scientists and to artists, thereby profoundly constraining the participating sets of expertise in cross-disciplinary projects.
- *Collaboration and connectivity*: Most interdisciplinary research is premised on some model of collaboration or cross-fertilization (even if the patterning of discipline occurs within the work of one researcher); once interdisciplinary projects involve a number of individuals, the question of how to understand relationships between and across those individuals becomes central. Of course this raises conceptual and methodological questions: in which respects are relationships between collaborators egalitarian, hierarchical or something else entirely (Weinberg *et al.*, 2011)? What models of social ties are mobilized, explicitly or implicitly, when discussing or analysing collaboration? How best might we visualize the connections among collaborators—what are the criteria, in other words, through which one adjudicates that a “connection” or “collaboration” has formed? Hubbub includes researchers who have addressed such questions from different disciplinary perspectives—most notably through problematizing social scientific accounts of “the social” (Fitzgerald and Callard, 2015), and through reflecting on the difficulties of visualizing connectivity in the brain (Margulies *et al.*, 2013). When the Core Group first thought through the project, it assembled a dense network map of connections between the proposed collaborators (Margulies, 2015). Although only a starting point, and one that is undoubtedly preliminary in some of its categorizations of collaborators’ expertise, the map takes collaboration seriously as an empirical object and has motivated us to think much harder about social networks for re-imagining the topologies of collaboration—a project that we are taking forward via the Hubbub Collaborator Questionnaire, detailed below.

## Three techniques

The commitments outlined above make the “interdisciplinary collaboration” of Hubbub a distinctive proposition: through our experimental, spatial and connective ambitions, we insist that collaboration is not a platitude, a norm or a telos; instead, for us, it describes a distinctive and changeable set of practices, an object of enquiry, a field of dispositions, a relation of power, an intervention in a space, a set of affective and embodied comportments, and so on. This means that collaboration is something to be investigated *in itself*: we are, in other words, keen to subject collaboration to an enquiry, while also exposing particular techniques *of* enquiry (whether that of the survey, the network analysis, the practice of observation) to new, interdisciplinary pressures, to make strange the means of collecting data, as well as the objects of which these data speak.

### Ethnographic analysis

Amid a growing literature on the need for, impetus behind, or desire underlying “interdisciplinary projects” (a literature to which we have ourselves contributed—see, for example, Bernini and Woods, 2014; Fitzgerald and Callard, 2015), there has been strikingly little attention to what large-scale and complex interdisciplinary projects actually look like *in the making*. We have a fair sense of why we should (or should not) pursue interdisciplinarity; of how interdisciplinary research is, might and should be designed and organized (Aldrich, 2014; McLeish and Strang, 2014); of the (surprisingly contingent) formations of particular disciplines; of the dense histories of interdisciplinarity within different parts of the academy (Downing, 2012; Fontaine, 2015; Graff, 2015); of the intellectual, technological and political-economic landscapes that demand hybrid methods; of judgements about the extent of interdisciplinarity within different domains of scholarship (Leydesdorff, 2007; Rafols and Meyer, 2010); about the difficulties of instituting interdisciplinarity (Fuller *et al.*, 2012); of the (imagined) forms of decorum and comportment appropriate to being in an interdisciplinarity setting; and even of the (multiple, unstable) metaphysics of objects that seem to elude narrow disciplined thinking (Latour, 1988). And, yet, we still know remarkably little of the mundane detail of what it looks and feels like to labour in an interdisciplinary setting. Nor, more importantly, do we have much sense of the *consequences* of this unfolding, uncertain, hybrid and multiple science-and-humanities-and-arts-in-the-making for what is, in fact, increasingly coming to be understood as the basic (indeed, correct and proper) praxis of interdisciplinarity today.

There have been some early forays into this domain—notably, Rabinow and Bennett’s ethnographic account of their interdisciplinary experiment with synthetic biology (Rabinow and Bennett, 2012). Also in synthetic biology—which is unusual in building at least the desire for collaboration, and reflection on collaboration, into its basic practice—Balmer and Bulpin have explored the relations between the members of an undergraduate team producing novel microorganisms, and have shown how these relations were bound within a sharp winnowing of what ended up being understood as ethical, legal and social components of the process (Balmer and Bulpin, 2013; cf. Marris *et al.*, 2014). Elsewhere, one of us (Fitzgerald), with colleagues, has published an auto-ethnographic account of the kinds of mundane emotional labour and compromise that attended one small-scale attempt to put together an interdisciplinary neuroscientific experiment—which concluded, against the usual advice, that tongue-biting was as much a virtue as plain-speaking in the assemblage of interdisciplinary experiments (Fitzgerald *et al.*, 2014).

These are early—and valuable—contributions, but there remains no sub-field of ethnographic collaboration studies, and no sustained attention to the micro-practices of interdisciplinary working, despite the prominence of this norm within the contemporary academy. The relative paucity of this literature is striking *vis-à-vis* the usual attention to “science in the making” within the science and technology studies (STS) literature. At least since Latour and Woolgar’s *Laboratory Life* (Latour and Woolgar, 1986), an account (to put it crudely) of doing science at the Salk Institute in San Diego, much STS has taken place within the genre of “laboratory ethnography”—thereby proceeding on the assumption that to understand a scientific object, or a practice, means understanding mundane relations, actions, thoughts, guesses, conversations, jokes, things, failures and so on, as well as the forms of sight and attention, the subtle technological manipulations, and the implicit norms and relations that make up the daily life of scientific assemblage. In Hubbub, we are trying to bring this method—this form of attention—to collaboration itself.

To that end, the project team includes an in-house participant observer, Fitzgerald, who is tasked with paying an ethnographic and “meta” attention to the daily workings of the project itself. As part of his role, Fitzgerald will attend to significant parts of all of the project’s main wings, interview key participants in the project (ensuring that he includes the full range of academic disciplines, as well as technological and artistic practices), pay attention to the daily workings of the space of The Hub, and maintain a field diary of significant (and sometimes insignificant) events in the life of Hubbub. The hope is that, by the end of the project, we will have amassed a rich ethnographic archive, not on the ambitions and achievements of the project, but on what it was actually like to put together, and work within, a flagship interdisciplinary project, between 2014 and 2016.

This is not as straightforward as it may appear: like any large-scale collaborative project, Hubbub is not without its tensions, and these are not always intellectually or affectively trivial. Indeed, our conjecture is that such tensions are central to, though frequently disavowed within, interdisciplinary (and, no doubt, disciplinary) projects—Rabinow and Bennett’s account notwithstanding. Such tensions can arise, and have already arisen in Hubbub, over differing epistemologies, ontologies and presumed foci or objects of analytic concern (see also contributions to Barry and Born, 2013); pragmatic differences in the setting of priorities and appropriate outcomes; adjudications of scientific and aesthetic quality; and co-ordination of the spacing and timing of research, given that co-investigators and collaborators are frequently scattered in laboratories and research spaces in and beyond the United Kingdom. Tensions, it is important to emphasize, can and do serve as catalysts for creativity, even as they place affective and social constraints on directions of research travel. We are still in the process of determining what this would mean for an ethnography, *vis-à-vis* both our ethical responsibilities to the other co-investigators and collaborators, as well as to our funder and to other Hubbub stakeholders (not all of whom, quite reasonably, are keen for us to dwell on, if not to treat as potentially publishable research fodder, what commonly remains intimate and unarticulated knowledge held closely to individuals’ chests). It is not yet settled how much access to the inner workings of Hubbub the ethnographer or his various publics will be granted. These complex, weighted negotiations characterize the entirety of the project; indeed, it is difficult to imagine how such negotiations could definitely come to a stop before Hubbub itself.

### “In the Diary Room”

Our second experiment—one explicitly aesthetic as well as scientific—is designed to bring to visibility the affective dynamics of interdisciplinarity. We are developing a tool to gather verbal, gestural and affective data from collaborators, as they reflect on their experiences of interdisciplinarity, when they are physically present in The Hub. This experiment finds its initial inspiration from what might be described as the epistemologically dubious though culturally resonant *mise-en-scène* of “The Diary Room”, as featured in the television franchise Big Brother (Big Brother UK Wiki, n.d.). The diary room in Big Brother is a small room accessed via the larger space of the Big Brother house, where participants are summoned (or might venture of their own accord) to answer questions posed by a disembodied voice—for example, about how they are feeling, what they are thinking—as well as be given tasks to carry out. While we torque the conventions of this televisual form, our own interdisciplinary experiment does start from the following social facts: (1) there is a small room within the Hub (see Fig. 1) that is set apart from the main activity of The Hub and where the door can be closed to maintain privacy; (2) Hubbub’s location on the fifth floor of Wellcome Collection results in many quotidian encounters with Wellcome Trust staff, as well as instances of actual and imagined—at least on Hubbub’s part—observation of Hubbub researchers’ actions by those staff; (3) several Hubbub researchers’ desire to capture the ephemera and trash of everyday, interdisciplinary life in the Hub for retrospective consideration. We are therefore in the midst of implementing our own computerized, voice-recording “Diary Room” in The Hub, which we anticipate running until the end of the residency. Collaborators who consent to be part of this experiment will be randomly called to the room, where a machine featuring the disembodied voices of various Wellcome Trust staff will ask questions about interdisciplinarity, while recording the response. In addition to its use as a data-gathering exercise, the performative nature of this experiment is intended to stage precisely that mix of playfulness, anxiety over surveillance, confessional affective logics and the turning inside-out of what “matters” in capturing a project, that is at the heart of Hubbub’s commitment to collaboration as such.

### The Hubbub Collaboration Questionnaire (HCQ) evaluative survey

Many empirical investigations of interdisciplinarity centre on the efficacy of teams in professional practice (for example, Nancarrow *et al.*, 2013). Studies in health-care settings, for example, tend to portray interdisciplinary working as being oriented towards specific goals, conducted through highly regulated protocols, predicated upon specialized and clearly delineated roles and institutionally mandated because it is regarded as essential to getting the job done. There is, indeed, a great premium placed on such successful team working in light of the exigencies of patient care (for example, Hamman, 2004). Academic research settings, particularly those involving humanities, arts and social science scholars, by contrast, constitute a different context for the study of interdisciplinarity in action: outcomes are, perhaps of necessity, more open-ended; methods can be more exploratory, especially where teams are newly constituted around specific projects; and roles are likely to be more varied, fluid and open to negotiation. (For one example of this, see “Working Knowledge” (Fernyhough *et al.*, 2015), which documents practices of working together in the interdisciplinary medical humanities project “Hearing the Voice”, and which gives several examples of exploratory working by members of a newly constituted team.)

The HCQ allows us to map these dynamics quantitatively and qualitatively as they play out across the project. Inspired by an online survey that was developed within the interdisciplinary “Hearing the Voice” project (Robson *et al.*, 2015), the HCQ consists of 18 fixed-choice and open-ended questions exploring people’s experiences of being part of an interdisciplinary team, as well as a separate scale in which each participant assesses the degree to which she sees a significant or meaningful degree of intersection between her own practice and those of other collaborators. The HCQ is being used at two time points across the 22-month project—at 6 and 18 months—to capture change across time. Ethical approval was received from the Department of Psychology at Durham University, and data from time point 1 are currently being analysed.

The HCQ uses a range of quantitative measures to capture, probe and interrogate the diverse profiles and experience of collaborators. Participants are invited to select the best description of their role (for example, “academic—doctoral researcher”, “journalism/media”) and expertise (for example, “creative arts—visual”, “science—neuroscience”), to indicate the way(s) they have engaged with the project (for example, “I attended the Hub Launch”, “I have spent time working in the Hub, mostly with others”) and to assess their degree of intersection with other collaborators. These data—differentiated by disciplinary identity and mode of engagement—will additionally enable us to conduct a social network analysis of individuals’ perceptions of their connectedness to other collaborators. This, in turn, can be analysed alongside any future outputs and outcomes of the project and will contribute to the small and growing body of literature using social network analyses to explore interdisciplinarity within teams (for example, Ryan *et al.*, 2014). While we expect to see little variation in participants’ reporting of their expertise, we anticipate (and, indeed, hope) that the picture of collaborator connections will change significantly between the two time periods, offering a much richer insight into the dynamic topologies of collaboration than any list of project outputs that we will compile when our residency in The Hub has ended in 2016.

Complementing these quantitative measures, several open-ended questions solicit people’s reflections on the temporal and spatial dimensions of interdisciplinary collaboration. We asked participants to describe their experiences of interdisciplinary working, expectations of Hubbub and of their role within the project, to reflect on whether and if so how these have changed, and to rank three things they would like to get out of their collaboration. Space is, as we have already noted, integral to the identity and operation, as well as the research interests, of the Hubbub project. The large, open-planned physical space of the Hub has its digital analogue in the “Slack” online project platform;^2^ the project is uniquely situated to afford multiple points of (swipe-card or password-protected) access, and to facilitate various modes of engagement. So how is participation in the project influenced by, and reflected in, collaborators’ use of these dedicated project spaces? Does The Hub itself function as an office, a studio, a university outpost? At what other sites is the work—and play—of the project undertaken? These are the questions that the HCQ burrows into.

## Conclusion

We have provided a schematic outline of our efforts to map the temporally and spatially complex practices of interdisciplinarity within Hubbub and The Hub at Wellcome Collection. Our approach is one that torques existing methods and conventions of evaluation and process-tracking. In so doing, we hope to engage playfully with the traditions and techniques of a number of disciplines on which Hubbub itself draws, as well as acknowledge the geographical distinctiveness of our current relationship to our funder, which is rather more physically and affectively entangled than is the case with many other interdisciplinary projects.

This sense of distinction also makes us slow to draw general conclusions, from our experience, that others might thereby apply to their own interdisciplinary labours. Nonetheless, there are (tacit) themes guiding our approach that we think could be profitably applied elsewhere: (1) Collaboration is not a goal to aim for, but an historically and culturally specific mode of practice that needs to be constantly interrogated, expanded and torqued. This means that serious, thoughtful self-tracking and self-analysis should be at the heart of major collaborative projects. This is something different from evaluation and project management: high-quality modes of tracking and tracing should be central to interdisciplinary endeavours, and should fold back onto the central questions *of* the project, as the work progresses. (2) Longstanding, trusted methods for self-reflection play a role (and we put both our questionnaire and ethnography in this bracket)—but it is important to be open, too, to the experimental, the playful and even the casual. The dynamics of interaction in collaborative spaces are often most visible in odd, serendipitous moments, not well-captured through orthodox social science methods. Our “In the Diary Room” is our attempt to generate something different—but doubtless there are many more ideas that might be employed. (3) Variegated spatial and temporal dynamics constitute the collaborative infrastructure of interdisciplinary projects—and those wishing to understand such projects should be attentive to them. We have been intensely interested in how collaborators use the Hub space, in the deployment of its different dimensions, in the temporal determinants of people’s physical presence, in the relationship between our project and the distance of its different sites, as well as the time it takes to close those differences and so on. We advise anyone interested in understanding projects like this one to put time and space at the centre of their investigation. (4) Not all collaborative projects are the same. One of the many elements that (we hope) distinguishes Hubbub is its lively interest in novel dynamics of experimentation—which is an interest that we both attend to in our tracking projects, but that has also informed how we design those tracking devices themselves. Other projects might similarly think through what kinds of iterative relationships might exist between the distinctive elements of their *own* collaboration, and the means through which they try to bring its progress into understanding.

Future publications from Hubbub will present, analyse and visualize data emerging from these three experiments—yielding, we hope, a less sanitized and domesticated account of interdisciplinarity than some of the current endorsements that characterize the field (for example, Allmendinger *et al.*, n.d.). Interdisciplinarity is necessarily and irrevocably a practice that entwines bodies, minds, geographies and temporalities in creative, ambivalent and often conflictual ways: the point of tracking the signal and tracing the noise of its explicit and not-so-explicit contours is precisely to do justice to these dynamics.

## Notes

1 In many parts of the natural sciences, the contingency of disciplines has long been to the fore (via, for example, the emergence of such assemblages as biochemistry and neuroscience; see, for example, Rees and Rose, 2004), while in the humanities the development of cross-disciplinary fields, especially those that *cross* into the natural sciences, such as the digital or medical humanities (see, for example, Greco, 2013), remains recent and novel.

2 “Slack” is an online team communication tool that provides topic-based channels, as well as private groups and direct messaging (see https://slack.com).
